# Impact of COVID‐19 infection on cognition and its association with neurological symptoms

**DOI:** 10.1002/brb3.2902

**Published:** 2023-02-21

**Authors:** Marta Almeria, Juan Carlos Cejudo, Jose Sanz‐Santos, Joan Deus, Jerzy Krupinski

**Affiliations:** ^1^ Medicine Department Autonomous University of Barcelona Barcelona Spain; ^2^ Department of Neurology Hospital Universitari MútuaTerrassa Terrassa Barcelona Spain; ^3^ Cognitive Impairment and Dementia Unit, Hospital Sagrat Cor Hermanas Hospitalarias, Martorell Barcelona Spain; ^4^ Department of Pneumology Hospital Universitari MútuaTerrassa Terrassa Barcelona Spain; ^5^ Clinical and Health Department, Psychology Faculty Autonomous University of Barcelona Barcelona Spain; ^6^ MRI Research Unit, Hospital del Mar Barcelona Spain; ^7^ Healthcare sciences Manchester Metropolitan University, CBS Manchester UK

**Keywords:** cognitive impairment, COVID‐19, neurologic symptoms, neuropsychology

## Abstract

**Objective**: To characterize the cognitive profile following COVID‐19 infection and its possible association to clinical symptoms, emotional disturbance, biomarkers, and disease severity.

**Methods**: This was a single‐center cross‐sectional cohort study. Subjects between 20‐ and 60‐year old with confirmed COVID‐19 infection were included. Evaluation was performed between April 2020 and July 2021. Patients with previous cognitive impairment and other neurological or severe psychiatric disorders were excluded. Demographic and laboratory data were extracted from the medical records.

**Results**: Altogether 200 patients were included, 85 subjects were female (42.3%), and mean age was 49.12 years (SD: 7.84). Patients were classified into four groups: nonhospitalized (NH, *n* = 21), hospitalized without intensive care unit (ICU) nor oxygen therapy (HOSP, *n* = 42), hospitalized without ICU but with oxygen therapy (OXY, *n* = 107), and ICU (ICU, *n* = 31) patients. NH group was younger (*p* = .026). No significant differences were found in any test performed attending severity of illness (*p* > .05). A total of 55 patients reported subjective cognitive complaints (SCC). Subjects with neurological symptoms (NS) performed worse in trail making test B (*p* = .013), digits backwards (*p* = .006), letter&numbers (*p* = .002), symbol digit modalities test (*p* = .016), and Stroop color (*p* = .010) tests.

**Conclusions**: OXY patients and females referred more SCC associated with symptoms of anxiety and depression. Objective cognitive performance was unrelated to SCC. No cognitive impairment was found regarding the severity of COVID‐19 infection. Results suggest that NS such as headache, anosmia, and dysgeusia during infection were a risk factor for later cognitive deficits. Tests assessing attention, processing speed, and executive function were the most sensitive in detecting cognitive changes in these patients.

## Graphical abstract

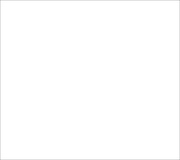

## 1 INTRODUCTION

Acute respiratory syndrome coronavirus 2 (SARS‐CoV‐2) principally targets the respiratory tract. However, there is growing evidence that COVID‐19 can also affect the central nervous system (CNS) (Li et al., 2020; Romero‐Sánchez et al., 2020) and cause CNS injury (Helms et al., 2020). Douand et al. (2022) identified significant longitudinal effects in brain imaging showing a greater reduction in grey matter thickness and tissue‐contrast in the orbitofrontal cortex and parahippocampal gyrus, changes in markers of tissue damage in regions functionally connected to the primary olfactory cortex and greater reduction in global brain size. The COVID‐19 participants also showed on average larger cognitive decline between the two time points. Subjective cognitive complaints (SCC) are among the most frequent neurological symptoms (NS) reported by patients following the acute infection (Bliddal et al., 2021). Those who suffered from COVID‐19 may complain of cognitive dysfunction (Almeria et al., 2020) often described as brain fog. The presence of neuropsychological deficits following SARS‐CoV‐2 is likely to result from multiple and interacting causes, such as a direct damage by the virus to the cortex and adjacent subcortical structures, or from psychological trauma (Ritchie et al., 2020).

Heterogeneous findings were reported in several cognitive domains, specifically in attention and executive function (Almeria et al., 2020; Altuna et al., 2021; Daroische et al., 2021; García‐Sánchez et al., 2022; Hadad et al., 2022; Hampshire et al., 2021; Woo et al., 2020; Zhou et al., 2020). Boesl et al. (2021) found out that 30% of patients with SCC had pathological scores in the Montreal Cognitive Assessment test (MoCA). Crivelli et al. (2022) in a systematic review and meta‐analysis found that patients recovered from COVID‐19 had lower general cognition in MoCA test compared to healthy controls. Daroische et al. (2021) demonstrated that the percentage of patients with global cognitive impairment ranged from 15% in Van Den Borst et al. (2021) to 80% in Alemanno et al. (2021). A study of 279 hospitalized patients found that 34% patients reported memory loss and 28% impaired concentration approximately 3 months after the discharge (Garrigues et al., 2020). Deficits in executive functioning, processing speed, category fluency, and memory encoding were also found (Ariza et al., 2022; Becker et al., 2021). Other studies linked the relation between cognitive deficits and NS (Almeria et al., 2020; Guo et al., 2022). Beaud et al. (2021) did not find a correlation between cognitive scores and mechanical ventilation. Woo et al. (2020) did not find oxygen supplementations and pharmacological treatments to predict cognitive deficits. Other studies (Alemanno et al., 2021) found that patients who benefited from orotracheal intubation and ventilation had significantly better scores in attention compared to patients who received oxygen therapy with venturi masks. García‐Sánchez et al. (2022) found that hospitalized patients had significantly lower performance in the MoCA test and in processing speed than nonhospitalized (NH) patients but hospitalization did not have a significant effect on test performance in most domains. Conflicting results in several studies make it difficult to conclude with certainty how oxygen therapy/mechanical ventilation can prevent or worsen cognitive impairment.

Mazza et al. (2021) observed a high rate of cognitive deficits at 1 and 3 months, irrespective of medical severity of the illness, with just 22% of the sample showing a good performance in all domains. Executive function and psychomotor coordination were the most involved domains, followed by information processing, verbal fluency, and working memory. These effects were influenced both by the presence of psychopathology and by the systemic inflammation, confirming connection among depression, inflammation, and cognition. Hellgren et al. (2021) reported that, in some individuals, COVID‐19 infection may have a negative impact on cognition that lasts at least several months after discharge with immediate and delayed memory being the indices with scores below the cutoff points. Mattioli et al. (2021) did not support the presence of cognitive impairment in a selected population of COVID‐19 patients studied 4 months following the diagnosis, although they did not include patients that required oxygen therapy or intensive care unit (ICU) care. Anxiety, stress, and depression resulted to be significantly higher in COVID‐19 patients than in controls. When considering ICU patients, those had a higher susceptibility of developing cognitive impairment than mild cases (Mattioli et al., 2022).

Hadad et al. (2022) found that disease severity, premorbid condition, pulmonary function test, and hypoxia did not contribute to cognitive performance. In addition, there is not a clear link between the severity of the infection and the degree of cognitive impairment (Houben & Bonnechère, 2022).

High rates of psychological symptoms such as anxiety, depression, post‐traumatic stress disorder (PTSD), and/or suicidal behavior were reported in general population irrespective of infectious status following previous coronavirus epidemics (Jeong et al., 2016). Rogers et al. (2020) meta‐analysis found that after recovery from the SARS and MERS infection, sleep disorders, traumatic memories, emotional lability, fatigue, and impaired concentration/memory were reported in more than 15% of the patients at the follow‐up period (6 weeks to 39 months). Our previous study following acute COVID‐19 infection associated SCC with anxiety and depression (Almeria et al., 2020). Whiteside, Basso et al. (2022) and Whiteside, Naini et al. (2022) suggested that psychological distress was prominent in patients with acute sequelae after COVID‐19 infection and related to objective cognitive performance, but objective cognitive performance was unrelated to cognitive complaints. Moreover, Whiteside, Basso et al. (2022) and Whiteside, Naini et al. (2022) found 6 months after infection that psychological distress, particularly somatic preoccupation, and depression were the most frequently reported symptoms in these participants. In this line, studies about the mental status of COVID‐19 patients showed the presence of depression, anxiety, and PTSD (Guo et al., 2020). De Lorenzo et al. (2020) study found that a quarter of their patients presented cognitive impairment in MoCA and 22.2% developed PTSD. Amanzio et al. (2021) studied the association among cognitive, physical, and behavioral prior, during and after the lockdown measures in cognitively normal aging subjects and found out that fatigue was related to mood deflections and cognitive function in terms of psychomotor speed. During and following the infection, patients were at increased risk to develop depression and anxiety symptoms (Deng et al., 2021) suggesting that psychological factors and other persisting symptoms such as fatigue and sleep disorders may play a significant role in SCC (Ceban et al., 2022; Krishnan et al., 2022).

Almost 2 years after the COVID‐19 outbreak, there is growing evidence of its impact on cognitive performance. Most studies did not consider emotional functioning or addressed performance validity as well as they used small samples of patients or mainly brief cognitive test or online surveys, which are not suitable to characterize the neuropsychological profile associated with COVID‐19 (Daroische et al., 2021). Sustained subclinical neuropsychological impairment could be a common sequel after COVID‐19 in young adults (Woo et al., 2020) suggesting that COVID‐19 could leave cognitive and emotional dysfunctions, whose underestimation may be costly in terms of long‐term morbidity and mortality. Therefore, neurologist and neuropsychologist are facing an increasing number of requests for assessment and treatment of patients with cognitive squeals after COVID‐19 infection (Sozzi et al., 2020). An early detection of neuropsychological manifestations and its possible association with clinical features and blood biomarkers may modify the risk of developing irreversible impairment and cognitive decline over time. Tracking the impact of COVID‐19 on cognitive and psychological patient conditions has relevant implications for rehabilitation strategies and long‐term assistance. The aim of this study is to characterize the clinical and neuropsychological manifestations and to report the SCC in the subacute period following COVID‐19 infection.

## 2 METHODS

### 2.1 Study design and participants

This is a consecutive case series cross‐sectional study that included adult patients evaluated in a universal and free nationalized health care hospital at Hospital Universitari MútuaTerrassa (HUMT) from April 2020 to July 2021. All patients included in the study had SARS‐CoV‐2 infection confirmed by positive polymerase chain reaction from nasopharyngeal swab or by positive serology. Patients were between 20‐ and 60‐year old. Subjects over 60 years of age were excluded to avoid age‐related cognitive decline. Patients with previous cognitive impairment and any other manifestation of the CNS or sever psychiatric disorders with potential cognitive deficits were also excluded. None of the participants were scheduled for disability scheme. The assessment was performed between 10 and 34 days post hospital or ambulatory discharge. The study was approved by the local ethic committee and all subjects signed the informed consent.

### 2.2 Data collection and definitions

Data was collected from the HUMT database, and a retrospective review of the electronic health records was performed. Demographic data, underlying comorbidities, and blood examinations that included ferritin and d‐dimer, symptoms and signs at presentation, and previous cognitive impairment were collected and evaluated. Clinical outcomes included length of stay, length of symptoms, need for the invasive mechanical ventilation and discharge disposition. Cognitive complaints were examined on the same day of neuropsychological assessment through an open question to the participant asking if they had noticed any cognitive change after COVID‐19 infection. To assess cognitive impairment, a set of subtests were selected to create a neuropsychological battery specific for this population. Neuropsychological evaluations were performed by the same expert in neuropsychology in 1 h session. All tests were validated in our population and are used internationally. The battery included the Test de Aprendizaje Verbal España‐Complutense (TAVEC) (Benedet & Alejandre, 2014), Visual Reproduction of the Wechsler Memory Scale IV (WMS‐IV) (Weschler, 2012), digits forward and backward, letter&numbers, trail making test A and B (TMT), symbol digit modalities test (SDMT), Stroop, phonemic and semantic fluency, and Boston Naming Test from the NEURONORMA project (NN) (Peña‐Casanova, Gramunt‐Fombuena, et al., 2009; Peña‐Casanova, Quiñones‐Ubeda, Gramunt‐Fombuena, Aguilar, et al., 2009; Peña‐Casanova, Quiñones‐Ubeda, Gramunt‐Fombuena, Quintana, et al., 2009; Peña‐Casanova, Quiñones‐Ubeda, Gramunt‐Fombuena, Quintana‐Aparicio, et al., 2009; Peña‐Casanova, Quiñones‐Ubeda, Quintana‐Aparicio, et al., 2009; Tamayo et al., 2012). The scores used for the analysis were the standardized notes, according to normative data in our environment, thus correcting the effects of the subjects’ age and education, specifically used the *T* note (PT) (mean 50 points and SD of 10 points). The Hospital Anxiety and Depression Scale (HAD) (Terol‐Cantero et al., 2015) was administered to assess symptoms of anxiety and depression.

### 2.3 Statistical analysis

Sample data and cognitive results were described assuming normal distribution, knowing its performance in larger samples, and standardized in our population. Standardized punctuations (*T* scores) for different cognitive tests were expressed in frequencies as an expression of pathological results in those with scores equal to or less than 30 in their *T* score (corresponding to 2 SD or less). Inferential tests were performed to compare cognitive performance according to other characteristics of the sample of clinical relevance. Comparisons between cohorts were analyzed using analysis of variance (ANOVA), and Levene test was used to assume or not equal variances on groups of comparison analysis. Kruskal Wallis was used when inferential test did not follow requirement of number of patients for group.

Sample was divided according to severity of illness into four categories depending on the requirement of hospitalization, oxygen therapy, and ICU admission. Four groups were created: NH (*n* = 21), hospitalized, no ICU nor oxygen therapy (HOSP, *n* = 42), hospitalized no ICU but with oxygen therapy (OXY, *n* = 107), and ICU (ICU, *n* = 31). Regarding neuropsychological impairment groups were divided as pathologic when *T* scores were <30, inferior performance when *T* scores were between 30 and 39, normal‐inferior when *T* scores where between 40 and 49, and normal functioning when *T* scores were >50.

To correlate the NS of the disease in the acute phase with the possible effect on cognition, the presence or absence of the main symptoms was compared: fever, headache, anosmia, dysgeusia, diarrhea, fatigue, cough, skin affection, and myalgia in cognitive tests using Student's *t*‐test.

Finally, and looking at the relationship between the main NS on cognition (headache, anosmia, and dysgeusia), we studied whether the number of NS; 0, 1, 2, or all 3 had an influence on cognitive performance. For this purpose, the 4 groups (0, 1, 2 and 3 NS) were created, and the cognitive performances in all the tests were compared by analysis of covariance on the direct scores obtained using as co‐variables age and education.

Statistical analyses were performed using R. CRAN. *Oficina de software libre (CIXUG)*. Spanish National Research Network. http://cran.es.r‐project.org/.

## 3 RESULTS

### 3.1 Demographic and clinical characteristics

A total of 200 patients who tested positive for SARS‐CoV‐2 were included in the study. Demographic and clinical characteristics are described in Table 1 attending the severity group. Eighty‐five subjects were female (42.3%) with mean (SD) age of 48.58 (8.4) years, all subjects were Caucasian, 116 subjects were male (57.7%) with mean (SD) age of 49.53 (7.3), with no statistically significant differences (*t* = .847, *p* = .398). Mean (SD) age for education was 13.18 (4.07) years (range: 4–20). Mean (SD) value for d‐dimer was 1655.32 (1833.06) (range: 241.00–5568.00), mean (SD) value for ferritin was 955.07 (1258.47) (range: 21.20–5498.70). Laboratory findings showed that males had significantly higher levels of ferritin than females (mean [SD] 1595.49 [1174.58] vs. 645.29 [732.18] *t* = 6.66, *p* = .001) but not d‐dimer values (mean [SD] 1183.86 [1636.62] vs. 1047.20 [1150.92] *t* = .10, *p* = .913).

**TABLE 1:** Demographic and clinical characteristics of patients within different severity groups

**Characteristics**	**NH** mean (SD)	**HOSP** mean (SD)	**OXY** mean (SD)	**ICU** mean (SD)
Age, y	44.29 (11.36)	49.26 (7.24)	49.70 (6.73)	50.23 (6.73)
Scholarship, y	14.38 (3.26)	14.12 (4.16)	12.76 (4.13)	12.58 (4.04)
Hospital discharge, d	N/A	25.22 (8.19)	27.36 (7.42)	27.00 (8.85)
Hospitalization, d	N/A	5.14 (2.41)	8.83 (4.25)	18.48 (8.72)
d‐Dimer ng/mL	289.01 (150.42)	617.96 (314.42)	1166.74 (2316.54)	1945.87 (1695.57)
Ferritin ng/mL	110.23 (89.52)	698.35 (1013.03)	1248.27 (954.50)	1871.60 (1439.13)

Abbreviations: d, days; HOSP; hospitalized, not ICU, not oxygen; ICU, ICU required intensive care unit; NH, nonhospitalized; OXY, hospitalized, not ICU, oxygen; SD, standard deviation; y, years.

d‐Dimer was increased in ICU group in respect to HOSP (ANOVA *F* = 4.18 *p* = .017 Scheffé post hoc test *p* = .017 HOSP/ICU). Ferritin values were increased in ICU group regarding the two other hospitalized groups (ANOVA *F* = 10.73 *p* = .011 Scheffé post hoc test for HOSP/ICU *p* = .001, OXY/ICU *p* = .018), and statistically significance differences were observed in hospitalized not ICU groups (ANOVA *F* = 4.18 *p* = .017 Scheffé post hoc test *p* = .017 HOSP/OXY *p* = .021). Ferritin and d‐dimer were not related to cognitive impairment (*p* > .05).

The most common symptoms at onset of illness was fever (79 [39.5%]), fatigue (39 [19.5%]), cough (36 [18.0%]), headache (31 [15.5%]), and myalgias (8 [4%]). In the course of the infection, 189 patients (94.5%) had fever, 184 (92%) fatigue, 163 (81.5%) cough, 147 (73.5%) dyspnea, 142 (71%) headache, 129 (64.5%) myalgias, 115 (57.5%) dysgeusia, 116 (58%) diarrhea, 106 (53%) anosmia, and 28 (14%) skin affection. Thirty‐one patients (15.5%) required ICU and 138 (69%) required oxygen.

Attending severity illness group, NH groups were younger (ANOVA *F* = 3.162, *p* = .026). No differences were observed in years of education (ANOVA *F* = 1.98, *p* = .117). There were more females in the NH group (70%) (*χ* ^2^ *p* = .001), 52% in the HOSP group, 36% in OXY, and 29% in ICU. Days of hospitalization were longer in ICU group but differences were significant in all the three hospitalized groups (ANOVA *F* = 75.13, *p* = .001 Scheffé post hoc test *p* = .001 in all pairs of comparison). There were no differences between groups according to illness severity for HAD anxiety (*F* = 1.96, *p* = .121) or HAD depression (*F* = 1.15, *p* = .330).

### 3.2 Neuropsychological findings

Neuropsychological characteristics are described in Table 2. The scores for each test are expressed in *T* score. No significant differences were found in any test performance among the four groups attending severity of illness (ANOVA *F* between .04 and 2.34 Sig > .5).

**TABLE 2:** Neuropsychological outcomes within different severity groups

**Characteristics**	**NH** mean (SD)	**HOSP** mean (SD)	**OXY** mean (SD)	**ICU** mean (SD)
TAVEC‐1 (PT)	48.50 (9.33)	46.66 (7.54)	45.70 (9.12)	45.48 (8.88)
TAVEC‐5 (PT)	54.50 (7.59)	51.90 (8.62)	53.08 (9.94)	50.64 (9.63)
TAVECTotal (PT)	52.50 (7.86)	50.71 (6.76)	50.84 (8.14)	47.74 (7.62)
TAVEC‐B (PT)	43.00 (8.01)	47.38 (9.12)	44.39 (7.79)	44.83 (9.26)
TAVEC‐IMR (PT)	51.00 (8.52)	49.52 (8.24)	49.43 (9.09)	49.03 (9.78)
TAVEC‐IMRSC (PT)	53.00 (9.23)	49.04 (8.49)	50.46 (10.31)	49.66 (8.50)
TAVEC‐DFR (PT)	51.00 (8.52)	49.76 (8.69)	51.21 (10.96)	49.35 (10.93)
TAVEC‐DFRSC (PT)	51.50 (7.45)	50.00 (9.37)	51.30 (10.28)	50.00 (12.38)
TAVEC‐REC. (PT)	52.50 (7.86)	52.61 (8.57)	52.99 (7.79)	52.66 (7.84)
WMS‐IMR (PT)	46.12 (6.85)	47.20 (7.65)	47.10 (6.89)	45.16 (7.38)
WMS‐DFR (PT)	48.62 (7.96)	49.94 (7.87)	48.57 (7.64)	48.54 (6.44)
Digits forward (PT)	48.87 (7.71)	48.33 (6.33)	47.83 (7.41)	46.04 (7.03)
Digits backwards (PT)	50.25 (5.89)	47.85 (7.62)	47.33 (6.83)	47.50 (5.88)
Letter&numbers (PT)	46.00 (7.18)	46.25 (5.66)	46.69 (7.37)	44.67 (5.69)
TMT‐A (PT)	46.50 (8.67)	47.02 (8.13)	46.44 (8.46)	45.80 (7.53)
TMT‐B (PT)	44.87 (6.95)	43.98 (7.15)	43.42 (7.62)	42.32 (7.11)
SDMT (PT)	44.37 (6.92)	43.86 (7.09)	43.27 (6.66)	43.22 (5.33)
Stroop lecture (PT)	43.12 (8.18)	46.13 (7.39)	44.83 (7.76)	44.35 (7.41)
Stroop color (PT)	41.87 (6.87)	44.16 (7.17)	44.32 (6.25)	43.16 (6.39)
Stroop int. (PT)	42.75 (7.90)	45.00 (7.94)	44.33 (7.35)	44.08 (7.49)
Semantic fluency (PT)	47.50 (5.38)	49.40 (5.99)	47.68 (8.71)	46.93 (7.57)
Phonemic fluency (PT)	44.00 (6.55)	44.64 (6.28)	43.31 (6.76)	43.06 (6.94)
FCRO copy (PT)	58.00 (9.34)	53.72 (12.65)	52.34 (9.68)	50.80 (8.85)
BNT (PT)	47.37 (9.88)	47.08 (7.52)	47.00 (7.85)	45.16 (6.18)
HAD anxiety (PD)	9.05 (4.17)	6.69 (4.66)	6.65 (4.25)	6.45 (3.75)
HAD depression (PD)	6.05 (4.48)	4.57 (4.00)	4.49 (3.80)	4.10 (3.39)
Cognitive reserve (PD)	12.75 (2.86)	14.69 (5.01)	12.95 (4.72)	12.89 (3.98)

Abbreviations: NH, nonhospitalized; HOSP, hospitalized, not ICU, not oxygen; OXY, hospitalized, not ICU, oxygen; ICU, ICU required intensive care unit; TAVEC‐1, Test de Aprendizaje Verbal España‐Complutense learning 1; TAVEC‐5, Test de Aprendizaje Verbal España‐Complutense learning 5; TavecTotal, Test de Aprendizaje Verbal España‐Complutense sum of learning; TAVEC‐B, Test de Aprendizaje Verbal España‐Complutense learning B; TAVEC‐IMR, Test de Aprendizaje Verbal España‐Complutense immediate recall; TAVEC‐IMRSC, Test de Aprendizaje Verbal España‐Complutense immediate recall semantic clue; TAVEC‐DFR, Test de Aprendizaje Verbal España‐Complutense deferred free recall; TAVEC‐DFRSC, Test de Aprendizaje Verbal España‐Complutense deferred free recall semantic clue; TAVEC‐REC, Test de Aprendizaje Verbal España‐Complutense recognition; WMS‐IMR, Visual Reproduction of the Wechsler Memory Scale IV immediate recall; WMS‐DFR, Visual Reproduction of the Wechsler Memory Scale IV deferred free recall; TMT‐A, trail making test A; TMT‐B, trail making test B; SDMT, symbol digit modalities test; BNT, Boston Naming Test; HAD, Hospital Anxiety and Depression scale; PT, *T* score; PD, direct score.

Of the total sample, 55 patients (27.5%) reported SCC. Overall, 20% were in the NH and HOSP group, followed by OXY with 47.27% and finally the ICU group with the less percentage of SCC with 12.72%, being just the OXY group statistically different from the other groups (*χ* ^2^ = 8.54 *p* = .036). Overall, 20.7% of the patients reporting SCC were male and 36.9% females (*χ* ^2^: 6.42, *p* = .011).

**FIGURE 1:**
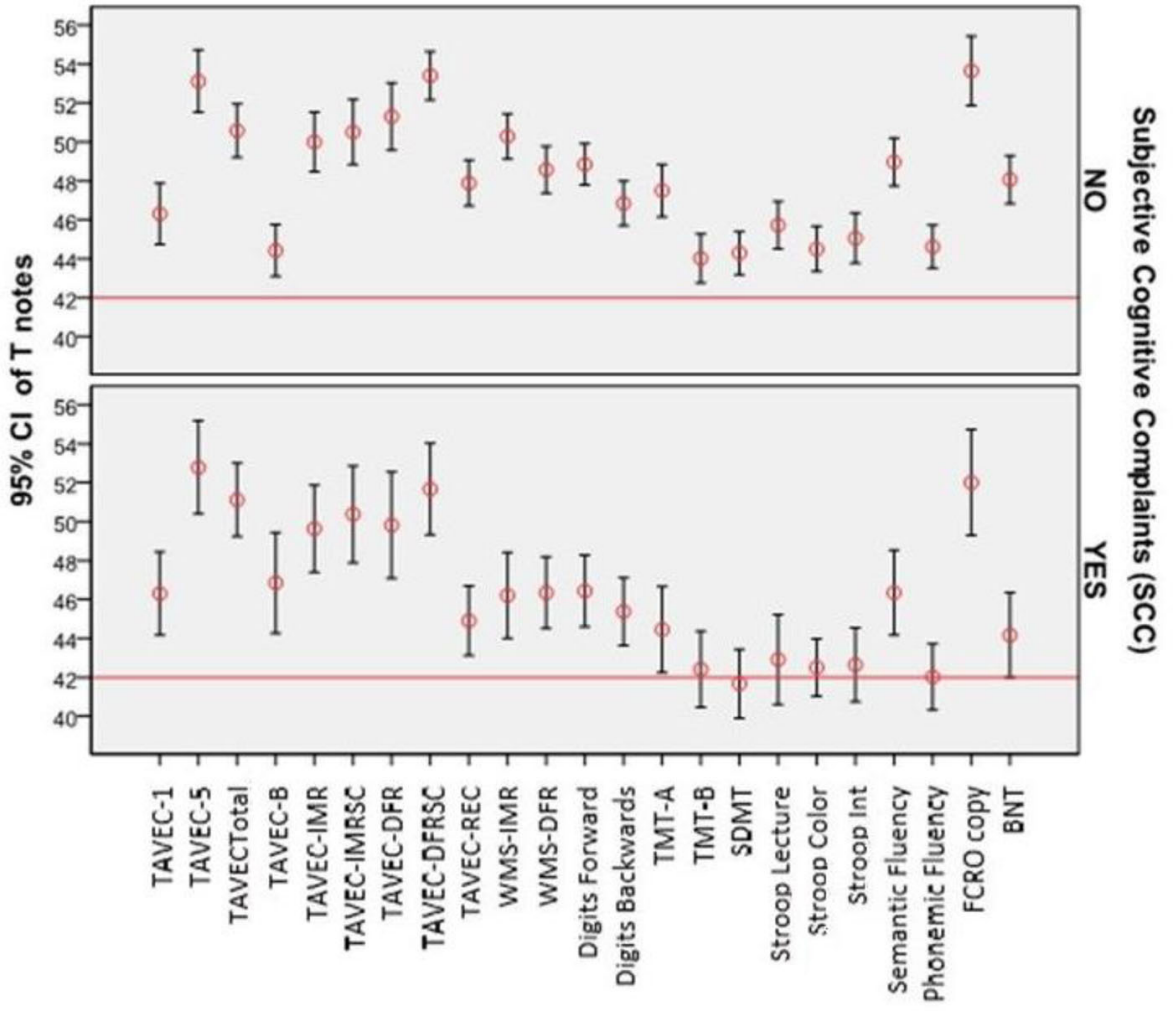
Cognitive performance according to subjective cognitive complaints. BNT, Boston Naming Test; SDMT, symbol digit modalities test; TAVEC‐1, Test de Aprendizaje Verbal España‐Complutense learning 1; TAVEC‐5, Test de Aprendizaje Verbal España‐Complutense learning 5; TAVEC‐B, Test de Aprendizaje Verbal España‐Complutense learning B; TAVEC‐DFR, Test de Aprendizaje Verbal España‐Complutense deferred free recall; TAVEC‐DFRSC, Test de Aprendizaje Verbal España‐Complutense deferred free recall semantic clue; TAVEC‐IMR, Test de Aprendizaje Verbal España‐Complutense immediate recall; TAVEC‐IMRSC, Test de Aprendizaje Verbal España‐Complutense immediate recall semantic clue; TAVEC‐REC, Test de Aprendizaje Verbal España‐Complutense recognition; TavecTotal, Test de Aprendizaje Verbal España‐Complutense sum of learning; TMT‐A, trail making test A; TMT‐B, trail making test B; WMS‐DFR, Visual Reproduction of the Wechsler Memory Scale IV deferred free recall; WMS‐IMR, Visual Reproduction of the Wechsler Memory Scale IV immediate recall.

Subjects with SCC did not show differences in any cognitive test when considering severity of illness (Sig > .05 for Kruskal Wallis in all comparisons). Figure 1 shows cognitive performance according to SCC. Although no statistical differences were observed in anxiety and depression, subjects with SCC showed higher punctuations in both scales (HAD‐A: 10.07, SD: 3.99) versus (HAD‐A: 5.66, SD: 3.75) and in (HAD‐D: 7.71, SD: 3.79) versus (HAD‐D: 3.42, SD: 3.42).

Comparing the percentage of patients over the cutoff point for anxiety and depression symptoms on the HAD scale, the group without cognitive complaints had a 31% of subjects above the cutoff score in the anxiety scale over the 72% in the SCC group (*χ* ^2^ = 27.35, *p* = .001). Regarding depression scale, 11% of the subjects in the noncognitive complaints had scores above the cutoff score, whereas the SCC group had a 52% of the subjects (*χ* ^2^ = 37.85, *p* = .001).

**FIGURE 2:**
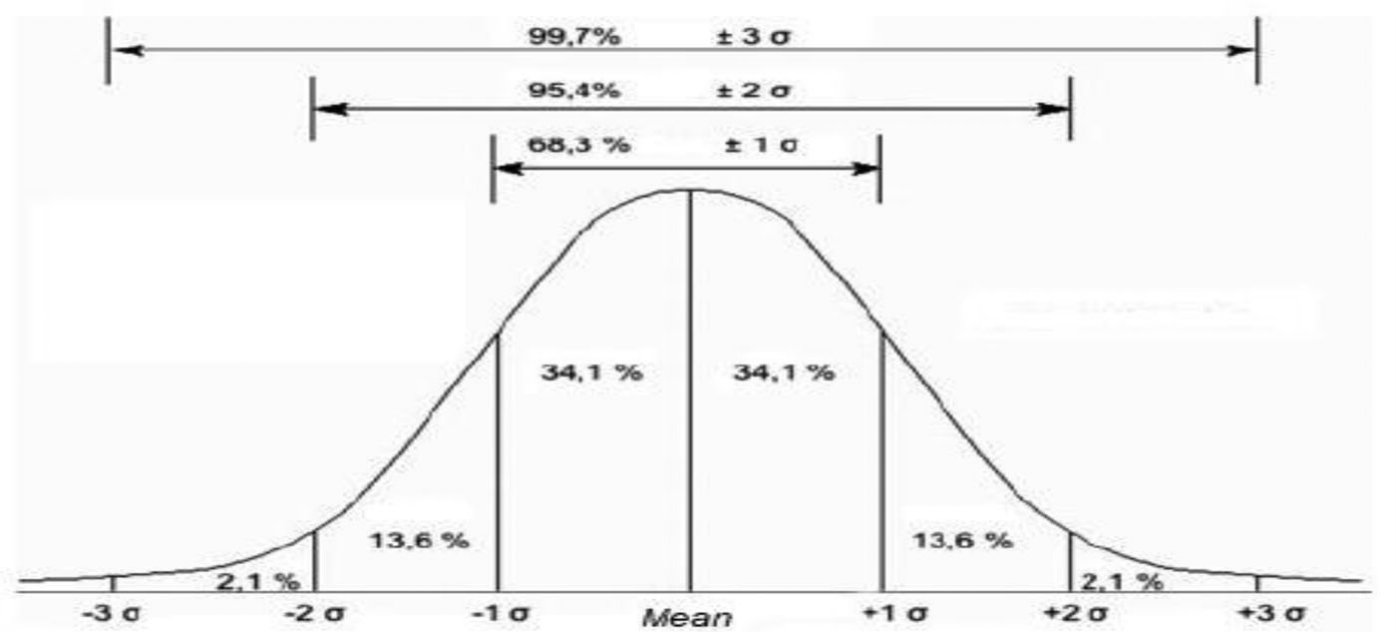
Percentage of subjects following normal distribution in our population for each of the neuropsychological tests within groups: with and without subjective cognitive complaints.

Figure 2 shows the curve for subjects following normal distribution. Table 3 shows the percentage of subjects for each cognitive subtest for all the sample and divided into groups (SCC vs. no complaints) and for each of the classifications based on neuropsychological performance (standard deviation below the mean). There is a tendency of a greater number of subjects in the first and second standard deviation below for subtests measuring learning, processing speed, attention, working memory, and executive function (TAVEC‐1, TAVEC‐B, WMS‐IMR, WMS‐DIF, SDMT, TMT‐A, Stroop, digits forward and backward, letter&number, semantic, and phonetic fluency) (see percentages marked in red in Table 3). There are no differences in percentages between patients with or without SCC.

**TABLE 3:** Percentage of subjects following normal distribution in our population for each of the neuropsychological tests within groups: with and without subjective cognitive complaints

**SD**	←3	−3	−2	−**1**	**+1**	**+2**	**+3**	**>+3**
**TAVEC‐1** All sample (%) NO complaints (%) Subjective cognitive complaints (%)	0.00 0.00 0.00	2.75 **3.45** 0.90	**25.50** **25.50** **25.45**	39.75 38.60 **42.75**	23.00 22.75 23.65	7.50 7.90 6.35	1.50 1.70 0.90	0.00 0.00 0.00
**TAVEC‐5** All sample (%) NO complaints (%) Subjective cognitive complaints (%)	0.00 0.00 0.00	2.00 2.40 0.90	9.75 9.65 10.00	26.75 26.90 26.35	36.75 36.20 38.15	21.25 20.70 22.70	3.50 4.15 1.80	0.00 0.00 0.00
**TAVECTotal** All sample (%) NO complaints (%) Subjective cognitive complaints (%)	0.00 0.00 0.00	1.50 2.05 0.00	10.25 11.00 8.20	35.00 34.10 37.30	39.00 38.25 40.90	13.50 13.80 12.70	0.75 0.70 0.90	0.00 0.00 0.00
**TAVEC‐B** All sample (%) NO complaints (%) Subjective cognitive complaints (%)	0.00 0.00 0.00	**4.25** **4.85** 2.75	**27.25** **27.95** **25.50**	**40.50** **41.40** 37.30	22.25 21.70 22.75	1.50 3.75 10.00	0.75 0.35 1.80	0.00 0.00 0.00
**TAVEC‐IMR** All sample (%) NO complaints (%) Subjective cognitive complaints (%)	0.00 0.00 0.00	2.25 2.75 0.90	15.50 15.50 15.45	33.25 32.75 34.55	46.75 32.10 34.55	14.50 13.50 14.55	1.00 1.40 0.00	0.00 0.00 0.00
**TAVEC‐IMRSC** All sample (%) NO complaints (%) Subjective cognitive complaints (%)	0.00 0.00 0.00	**3.50** **4.15** 1.80	12.55 12.50 12.70	31.40 30.25 34.55	34.90 35.10 34.55	15.05 15.65 13.65	2.50 2.45 2.75	0.00 0.00 0.00
**TAVEC‐DFR** All sample (%) NO complaints (%) Subjective cognitive complaints (%)	0.00 0.00 0.00	2.25 **3.45** 0.90	16.00 14.85 **20.90**	27.75 26.90 30.00	30.75 32.05 27.25	19.25 19.65 19.05	2.50 3.10 1.80	0.00 0.00 0.00
**TAVEC‐DFRSC** All sample (%) NO complaints (%) Subjective cognitive complaints (%)	0.00 0.00 0.00	**4.25** **4.85** 2.75	12.25 11.75 13.65	28.00 26.55 31.80	34.75 34.80 34.55	17.75 18.60 15.45	3.00 3.45 1.80	0.00 0.00 0.00
**TAVEC‐REC** All sample (%) NO complaints (%) Subjective cognitive complaints (%)	0.00 0.00 0.00	1.50 1.05 2.75	7.05 6.95 6.35	25.40 25.00 26.35	42.45 43.05 40.90	23.10 23.95 20.90	0.00 0.00 0.00	0.00 0.00 0.00
**WMS‐IMR** All sample (%) NO complaints (%) Subjective cognitive complaints (%)	0.00 0.00 0.00	1.25 1.40 0.90	19.75 17.95 **24.55**	**43.25** **39.35** **53.60**	35.00 40.00 19.95	0.75 1.40 0.90	0.00 0.00 0.00	0.00 0.00 0.00
**WMS‐DFR** All sample (%) NO complaints (%) Subjective cognitive complaints (%)	0.00 0.00 0.00	0.75 0.00 2.70	12.50 8.95 **21.75**	**41.75** **42.05** **40.80**	39.50 42.25 31.75	5.50 6.55 2.70	0.00 0.00 0.00	0.00 0.00 0.00
**Digits forward** All sample (%) NO complaints (%) Subjective cognitive complaints (%)	0.00 0.00 0.00	0.50 0.70 0.00	11.25 10.30 13.65	**50.75** **47.20** **60.05**	34.50 38.35 24.50	3.00 3.45 1.80	0.00 0.00 0.00	0.00 0.00 0.00
**Digits backwards** All sample (%) NO complaints (%) Subjective cognitive complaints (%)	0.00 0.00 0.00	1.50 1.05 2.75	9.25 8.70 10.9	**50.75** **48.30** **57.25**	35.50 38.50 27.30	3.00 3.45 1.80	0.00 0.00 0.00	0.00 0.00 0.00
**Letter&numbers** All sample (%) NO complaints (%) Subjective cognitive complaints (%)	0.00 0.00 0.00	1.25 1.05 1.80	12.50 12.20 11.60	**62.60** **61.10** **64.45**	21.80 20.50 16.45	3.00 5.25 1.80	0.00 0.00 0.00	0.00 0.00 0.00
**TMT‐A** All sample (%) NO complaints (%) Subjective cognitive complaints (%)	0.00 0.00 0.00	**7.00** **3.10** **4.55**	16.75 14.50 **22.80**	**49.50** **48.60** **51.80**	23.75 26.30 17.35	6.00 7.60 3.60	0.00 0.00 0.00	0.00 0.00 0.00
**TMT‐B** All sample (%) NO complaints (%) Subjective cognitive complaints (%)	0.00 0.00 0.00	**4.40** **3.85** **5.55**	**27.30** **25.15** **32.50**	**49.30** **50.05** **47.25**	18.05 19.10 14.90	1.25 1.75 0.00	0.00 0.00 0.00	0.00 0.00 0.00
**SDMT** All sample (%) NO complaints (%) Subjective cognitive complaints (%)	0.00 0.00 0.00	**3.00** **3.45** 1.80	**24.25** 17.90 **42.65**	**59.00** **62.70** **49.10**	12.25 15.95 4.55	1.00 0.00 1.80	0.00 0.00 0.00	0.00 0.00 0.00
**Stroop Lecture** All sample (%) NO complaints (%) Subjective cognitive complaints (%)	0.00 0.00 0.00	2.50 2.35 2.75	**23.05** 17.70 **37.30**	**49.15** **52.45** **40.00**	23.55 32.60 16.30	1.75 1.05 3.60	0.00 0.00 0.00	0.00 0.00 0.00
**Stroop color** All sample (%) NO complaints (%) Subjective cognitive complaints (%)	0.00 0.00 0.00	**3.00** **3.45** 0.90	**24.25** **21.70** **30.95**	**58.35** **57.40** **60.90**	14.30 17.15 7.25	1.00 1.40 0.00	0.00 0.00 0.00	0.00 0.00 0.00
**Stroop Int**. All sample (%) NO complaints (%) Subjective cognitive complaints (%)	0.00 0.00 0.00	**3.55** **3.50** **3.65**	**22.30** **20.65** **30.00**	**47.25** **44.45** **54.55**	25.25 30.45 11.70	0.75 1.05 0.00	0.00 0.00 0.00	0.00 0.00 0.00
**Semantic Fluency** All sample (%) NO complaints (%) Subjective cognitive complaints (%)	0.00 0.00 0.00	2.50 1.75 **4.50**	11.75 6.95 13.65	**46.25** **44.10** **51.75**	34.00 37.55 24.60	5.00 4.85 5.40	0.25 0.35 0.00	0.00 0.00 0.00
**Phonemic fluency** All sample (%) NO complaints (%) Subjective cognitive complaints (%)	0.00 0.00 0.00	**3.00** 2.75 **3.60**	**25.00** **23.75** **31.85**	**55.75** **55.15** **57.35**	14.25 16.95 7.20	0.75 1.40 0.00	0.00 0.00 0.00	0.00 0.00 0.00
**FCRO copy** All sample (%) NO complaints (%) Subjective cognitive complaints (%)	0.00 0.00 0.00	1.00 1.40 0.00	5.00 3.50 9.10	**41.25** **40.00** **44.50**	19.25 20.80 15.45	33.50 34.50 30.90	0.00 0.00 0.00	0.00 0.00 0.00
**BNT** All sample (%) NO complaints (%) Subjective cognitive complaints (%)	0.00 0.00 0.00	1.75 1.40 2.75	15.25 10.80 **27.40**	**52.25** **54.11** **47.20**	27.25 29.60 20.95	3.50 4.20 1.80	0.00 0.00 0.00	0.00 0.00 0.00

*Note*: All subjects (*N* = 200), no complaints (*N* = 145), cognitive complaints (*N* = 55).

Abbreviations: BNT, Boston Naming Test; HAD, Hospital Anxiety and Depression scale; PD, direct score; PT, *T* score; SDMT, symbol digit modalities test; TAVEC‐1, Test de Aprendizaje Verbal España‐Complutense learning 1; TAVEC‐5, Test de Aprendizaje Verbal España‐Complutense learning 5; TAVEC‐B, Test de Aprendizaje Verbal España‐Complutense learning B; TAVEC‐DFR, Test de Aprendizaje Verbal España‐Complutense deferred free recall; TAVEC‐DFRSC, Test de Aprendizaje Verbal España‐Complutense deferred free recall semantic clue; TAVEC‐IMR, Test de Aprendizaje Verbal España‐Complutense immediate recall; TAVEC‐IMRSC, Test de Aprendizaje Verbal España‐Complutense immediate recall semantic clue; TAVEC‐REC, Test de Aprendizaje Verbal España‐Complutense recognition; TavecTotal, Test de Aprendizaje Verbal España‐Complutense sum of learning; TMT‐A, trail making test A; TMT‐B, trail making test B; WMS‐DFR, Visual Reproduction of the Wechsler Memory Scale IV deferred free recall; WMS‐IMR, Visual Reproduction of the Wechsler Memory Scale IV immediate recall.

Table 4 shows difference for each subtest regarding clinical symptoms. Subjects with NS as anosmia, dysgeusia, and headache had worse performance in tests of processing speed, attention, and working memory than subjects without these symptoms. Considering NS, 29 (14.5%) did not have any, 56 subjects (28%) had one, 38 (19%) had two, and 77 (38.5%) had three NS. Statistical differences in cognitive performance attending NS were in TAVEC‐IMR (*F* = 3.79, *p* = .016), differences between subjects without NS and three NS (*p* = .027). Digits backward (*F* = 4.33, *p* = .006), difference between subjects without NS and three NS (*p* = .002). Letter&number (*F* = 5.21, *p* = .002), difference between subjects without NS and three NS (*p* = .004). TMT‐B (*F* = 3.69, *p* = .013) differences between subjects without NS and three NS (0.028). SDMT (*F* = 3.51, *p* = .016) differences between subjects without NS and three NS (*p* = .044). Stroop color (*F* = 3.90, *p* = .010) differences between subjects without NS and three NS (*p* = .009).

**TABLE 4:** Neuropsychological findings related to clinical symptoms

**NPS/symptoms**	**Anosmia^a^ (*N* = 106)**	**Dysgeusia^a^ (*N* = 115)**	**Headache^a^ (*N* = 142)**	**Fatigue (*N* = 184)**	**Cough (*N* = 163)**	Myalgia **(*N* = 129)**	**Fever (*N* = 189)**	**Diarrhea (*N* = 116)**
TAVEC‐1	NS	NS	NS	NS	NS	NS	NS	NS
TAVEC‐5	NS	NS	NS	NS	NS	NS	NS	NS
TAVECTotal	NS	NS	*p* = .035 *t* = 2.12	NS	NS	NS	NS	NS
TAVEC‐B	NS	NS	NS	NS	NS	NS	NS	NS
TAVEC‐IMR	NS	NS	*p* = .001 *t* = 3.33	NS	NS	NS	NS	NS
TAVEC‐IMRSC	NS	NS	NS	NS	NS	NS	NS	NS
TAVEC‐DFR	NS	NS	NS	NS	NS	NS	NS	NS
TAVEC‐DFRSC	NS	NS	*p* = .031 *t* = 2.17	NS	NS	NS	NS	NS
TAVEC‐REC	NS	NS	NS	NS	NS	NS	NS	NS
WMS‐IMR	NS	NS	*p* = .033 *t* = 2.15	NS	NS	NS	NS	NS
WMS‐DFR	NS	NS	*p* = .005 *t* = 2.86	NS	NS	NS	NS	NS
Digits forward	NS	NS	*p* = .004 *t* = 2.92	NS	NS	NS	NS	NS
Digits backwards	*p* = .024 *t* = 2.27	*p* = .007 *t* = 2.73	*p* = .025 *t* = 2.25	NS	NS	NS	NS	NS
Letter&numbers	*p* = .028 *t* = 2.20	*p* = .033 *t* = 2,14	*p* = .001 *t* = 3.27	NS	NS	NS	NS	NS
TMT‐A	NS	NS	NS	NS	NS	NS	NS	NS
TMT‐B	*p* = .006 *t* = 2.78	*p* = .007 *t* = 2.74	*p* = .013 *t* = 2.50	NS	NS	NS	NS	NS
SDMT	*p* = .001 *t* = 3.22	*p* = .006 *t* = 2.77	*p* = .013 *t* = 2.51	NS	NS	NS	NS	NS
Stroop lecture	*p* = .022 *t* = 2.30	*p* = .003 *t* = 2.35	*p* = .001 *t* = 3.36	NS	NS	NS	NS	NS
Stroop color	*p* = .001 *t* = 3.24	*p* = .003 *t* = 3.01	*p* = .002 *t* = 3.11	NS	NS	NS	NS	NS
Stroop int.	*p* = .038 *t* = 2.08	NS	*p* = .004 *t* = 2.89	NS	NS	NS	NS	NS
Semantic fluency	NS	NS	NS	NS	NS	NS	NS	NS
Phonemic fluency	NS	NS	NS	NS	NS	NS	NS	NS
FCRO copy	NS	NS	NS	NS	NS	NS	NS	NS
BNT	NS	NS	NS	NS	NS	NS	NS	NS
HAD‐A	NS	NS	NS	*p* = .002 *t* = 3.12	NS	NS	NS	NS
HAD‐D	NS	NS	NS	*p* = .001 *t* = 3.16	NS	NS	NS	NS

*Note*: values indicate differences between patients with symptoms and without symptoms. *N* represents subjects with symptoms. Non‐equal variances are assumed.

Abbreviations: BNT, Boston Naming Test; HAD, Hospital Anxiety and Depression scale. NS, No significance; NPS, neuropsychology; SDMT, symbol digit modalities test; Sig., Significance; *t*., Student Test; TAVEC‐1, Test de Aprendizaje Verbal España‐Complutense learning 1; TAVEC‐B, Test de Aprendizaje Verbal España‐Complutense learning B; TAVEC‐IMR, Test de Aprendizaje Verbal España‐Complutense immediate recall; TavecTotal, Test de Aprendizaje Verbal España‐Complutense sum of learning; TMT‐A, trail making test A; TMT‐B, trail making test B; WMS‐DFR, Visual Reproduction of the Wechsler Memory Scale IV deferred free recall.

**^a^** Neurological symptoms.

## 4 DISCUSSION

Our study was designed to characterize the extent of cognitive impairment and SCC in patients following COVID‐19 infection. In our cohort, fever was the predominant symptom in all patients, followed by cough, fatigue, and headache as described in previous studies (Almeria et al., 2020; Li et al., 2020). Males showed higher levels of ferritin than females (Bliddal et al., 2021) but not d‐dimer values. Both d‐dimer and ferritin were increased in ICU group, possibly due to its association with severity of illness.

We found that the neuropsychological performance profile of COVID‐19 patients, regardless of the degree of clinical severity, is similar to the normal population. Intriguing, hospitalization did not have a significant effect on test performance, and there was no statistical difference between NH patients, those who required hospitalization with or without oxygen and those that received ICU care. These results are consistent with previous reports of cognitive and neurological sequels 4 months after COVID‐19 infection (Mattioli et al., 2022) and where cognitive decline was independent of disease severity (Hadad et al., 2022; Houben & Bonnechère., 2022). Other studies also found no differences when considering treatment with mechanical ventilation or oxygen supply (Beaud et al., 2021; García‐Sánchez et al., 2022; Woo et al., 2020). Our results suggest that COVID‐19 infection per se does not appear to produce a great cognitive impairment. Cognitive deficits in our patients could be related to other factors such as previous unreported cognitive impairment or concomitant cerebrovascular diseases (Romero‐Sánchez et al., 2020), encephalopathy (Li et al., 2020; Xiang et al., 2020), and symptoms of anxiety and depression (Amanzio et al., 2021; De Lorenzo et al., 2020; Deng et al., 2021; Jeong et al., 2016; Rogers et al., 2020; Sozzi et al., 2020).

However, tests for learning ability, attention, processing speed, and executive function (TAVEC‐1, TAVEC‐B, WMS‐IMR, WSM‐DFR, digits Forward, digits backward, letter&number, TMT‐B, SDMT, Stroop, and semantic and phonetic fluency) were found in greater frequency than expected in the low normal range (below 1 or 2 SD) as compared to normal population. Our results are consistent with the observed in the literature where attention, memory, and executive function are the domains more affected in post COVID‐19 patients (Alemanno et al., 2021; Almeria et al., 2020; Ariza et al., 2022; Beaud et al., 2021; Becker et al., 2021; Daroische et al., 2021; García‐Sánchez et al., 2022; Hampshire et al., 2021; Van Den Borst et al., 2021; Woo et al., 2020; Zhou et al., 2020). As we did not have a prior to COVID‐19 infection neuropsychological assessment, our hypothesis is that although no cognitive impairment was found in our sample, subjects tend to appear in a lower performance range in those domains because there is a decline regarding their initial performance.

When analyzing our sample separately, that is, patients with SCC or without, both groups tend to stand in majority in a lower standard deviation. The fact that there are no differences between both groups could be explained that testing was very close to the recent infection, meaning all patients could still experience residual symptoms such as fatigue, anxiety, among others, that could affect cognitive performance. These results are similar to the ones found by Whiteside, Basso et al. (2022), Whiteside, Naini et al. (2022), and Ariza et al. (2022) where objective cognitive performance was unrelated with cognitive complaints. This is in‐line with our main finding that there is not enough evidence to support a clear consequence of a new disease in subjects in the acute period after COVID‐19 infection. However, we consider that there is a need to apply psychological and neuropsychological screening and intervention strategies when mild cognitive complaints and emotional disturbances appear in the context of recovery from COVID‐19.

Given that SARS‐CoV‐2 may enter the CNS through the hematogenous or retrograde neuronal route, supported by the fact that 106 (53%) of the patients in our sample had smell impairment, we also examined whether clinical symptoms could be associated with cognitive performance. NS such as headache, loss of smell, and taste were strongly associated with impairment in several subtests, including attention, memory, processing speed, and executive function (Almeria et al., 2020). Headache was the NS most associated with poor performance in neuropsychological test. Subjects with all three NS had worse performance in processing speed, attention, and working memory (TAVEC‐IMR, digits backwards, letter&number, TMT‐B, SDMT, and Stroop color), showing the association between NS and worsening performance. This phenomenon may be indicative of the potential of COVID‐19 for CNS invasion capacity in consonance with results showing the overlapping olfactory and memory‐related functions, including the parahippocampal gyrus/perirhinal cortex, entorhinal cortex, and hippocampus, which are frequent in SARS‐CoV‐2 (Douaud et al., 2022). As in our sample, infected participants had no signs of memory impairment but worsening in executive function, particularly in TMT‐B (Douaud et al., 2022). Our results also support the relation between fatigue and anxiety and depressive symptoms but not with cognition (Amanzio et al., 2021; Rogers et al., 2020).

Finally, we found that SCC were significantly higher in females and in the OXY patients group, probably associated with fear of awareness of the severity of the disease. Higher scores in anxiety and depression scales were reported, suggesting a greater impact of emotional wellbeing on SCC. Other studies have already pointed out the role of anxiety, depression, PTSD, and psychological distress in cognitive performance (Amanzio et al., 2021; Deng et al., 2021; Guo et al., 2020; Jeong et al., 2016; Krishnan et al., 2022; Rogers et al., 2020; Whiteside, Basso, et al., 2022; Whiteside, Naini, et al., 2022).

## 5 LIMITATIONS

One limitation of this study was the use of a retrospective chart review to collect clinical information and the absence of a standardized questionnaire to assess cognitive complaints. Another limitation was the use of a single self‐report measure for anxiety and depression symptoms. We encourage future studies to include more specific questionnaires to assess emotional functioning, neuropsychiatric symptoms, and PTSD. We analyzed the effect of the biomarkers that we were allowed to obtain as a routine in our setting. For that reason, another limitation is the lack of other specific inflammatory biomarkers which could be associated with systemic inflammation and not having information about the suspected COVID‐19 variant. The main limitation of this study was that we did not have any cognitive evaluation before COVID‐19 that could possibly show small differences from baseline and also the lack of a control group. We tried to minimize this limitation with the exclusion of older patients who might have had other concomitant pathologies that could affect cognition, such as vascular risk factors or incipient neurodegenerative diseases, as well as any previous neurologic or severe psychiatric disorder that might affect cognition. All test scores were corrected by standardized notes, according to normative data in our environment, thus correcting the effects of the subjects’ age and education. Future studies should address these limitations.

## 6 CONCLUSIONS

The findings in the current study allow us to characterize the cognitive profile of patients after COVID‐19 infection. Specifically, COVID‐19 patients can report SCC immediately after hospital discharge although no cognitive impairment was found when considering biomarkers or severity of illness. However, subjects tend to appear in a lower performance range specifically in test of processing speed, executive function, attention, and working memory. Additionally, having all three NS was indicative of worse performance in those domains. Clinicians should consider the presence of NS as a risk factor for cognitive worsening and neuropsychiatric symptoms such as anxiety and depression as a risk factor for SCC. A long‐term follow‐up is necessary to stablish the permanence of such complaints. Strategies that include psychological and cognitive rehabilitation especially in attention and executive function should be considered.

## CONFLICT OF INTEREST STATEMENT

M. Almeria reports no disclosures relevant to the manuscript. JC. Cejudo reports no disclosures relevant to the manuscript. J. Sanz‐Santos reports no disclosures relevant to the manuscript. J. Deus reports no disclosures relevant to the manuscript. J. Krupinski reports no disclosures relevant to the manuscript.

### PEER REVIEW

The peer review history for this article is available at https://publons.com/publon/10.1002/brb3.2902.

## Data Availability

All study data, including raw and analyzed data, and materials are available from the corresponding author on request.
